# Autoimmune-associated thrombosis: mechanisms, population burden, and prevention strategies

**DOI:** 10.1016/j.lanepe.2026.101850

**Published:** 2026-09-03

**Authors:** Deepa J. Arachchillage, Megan V. Preece, Mike Laffan

**Affiliations:** a Centre for Haematology, Department of Immunology and Inflammation, Imperial College London, London, W12 0NN, United Kingdom; b Department of Haematology, Imperial College Healthcare NHS Trust, London, W12 0NN, United Kingdom

**Keywords:** Autoimmune disease, Inflammation, Thrombosis, Cardiovascular events, Endothelial injury

## Abstract

Autoimmune diseases, including systemic lupus erythematosus, rheumatoid arthritis, antiphospholipid syndrome, and systemic vasculitis, are associated with increased risk of thrombosis, accelerated atherosclerosis, cardiovascular-events, and premature mortality. Heparin-induced thrombocytopaenia and vaccine-induced thrombocytopaenia and thrombosis (VITT) or VITT like syndrome are autoimmune thrombotic disorders that do not have additional features of autoimmune diseases. We summarise the epidemiology, pathophysiology, diagnosis, and management of autoimmune thrombosis. Key mechanisms include autoantibody-mediated coagulation activation, endothelial dysfunction, complement activation, platelet activation, neutrophil extracellular-trap formation, and thrombo-inflammation, promoting thrombin generation, impaired fibrinolysis, and vascular injury. Traditional cardiovascular risk factors, such as smoking, obesity, hypertension, diabetes mellitus, and infection, further amplify thrombotic risk. Management requires integrated strategies combining anticoagulation, immunomodulatory therapy, cardiovascular-risk reduction, and long-term surveillance. Autoimmune thrombosis is an under-recognised contributor to cardiovascular disease and premature mortality across Europe. Earlier diagnosis, improved risk stratification, multidisciplinary care, and integration of autoimmune diseases into European cardiovascular prevention and health-system strategies are essential to reduce morbidity and premature mortality.

**Funding:**

DJA is funded by Medical Research Council UK (MR/Z505274/1) and infrastructure support was provided by the NIHR Imperial Biomedical Research Centre.

**Key messages**

• Autoimmune-mediated thrombosis is an under-recognised contributor to premature cardiovascular disease, disability and mortality across Europe and globally, with important implications for patients, healthcare systems and population health.
• Chronic inflammation, autoantibody-mediated coagulation activation, endothelial dysfunction, complement activation and thrombo-inflammation drive both venous and arterial thrombosis and accelerate atherosclerosis across multiple autoimmune diseases.
• Antiphospholipid syndrome represents the archetypal autoimmune thrombotic disorder, but patients with systemic lupus erythematosus, rheumatoid arthritis, systemic vasculitis, and immune-mediated thrombotic syndromes such as HIT and VITT also have substantially increased thrombotic risk.
• Conventional cardiovascular risk prediction tools frequently underestimate vascular risk in autoimmune diseases. Earlier identification of high-risk patients and incorporation of autoimmune disease characteristics into cardiovascular risk assessment are needed.
• Cardiovascular prevention should be integrated into routine autoimmune disease management, combining optimal control of inflammation with aggressive management of traditional cardiovascular risk factors and appropriate antithrombotic therapy.
• Multidisciplinary care pathways linking primary care, rheumatology, haematology, cardiology and other specialities are essential to improve risk assessment, prevention and long-term outcomes.
• European health systems should recognise autoimmune diseases as cardiovascular risk-enhancing conditions and incorporate them into broader cardiovascular disease and non-communicable disease prevention strategies, clinical guidelines and quality improvement initiatives.
• Future priorities include harmonised European registries, autoimmune-specific cardiovascular risk prediction tools, implementation research, and equitable access to multidisciplinary preventive care to reduce avoidable cardiovascular morbidity, disability and premature mortality.

**Search strategy and selection criteria**

We conducted a literature search using PubMed/MEDLINE, Embase, and Web of Science until June 2026. However, recent publications (within last 10 years) were preferentially selected when multiple studies addressed similar topics. Search terms included Medical Subject Headings (MeSH) and free-text keywords related to “autoimmune thrombosis,” “autoantibodies”, “antiphospholipid syndrome,” “COVID-19 and thrombosis” “antiphospholipid antibodies,” “immunothrombosis,” “thromboinflammation,” “heparin induced thrombocytopaenia” “vaccine-induced immune thrombotic thrombocytopaenia,” “platelet factor 4 (PF4),” “venous thromboembolism,” “arterial thrombosis,” “neutrophil extracellular traps,” “NETosis,” “complement activation,” “biomarkers,” “prevention,” and “cardiovascular risk.” Additional articles were identified through manual searches of reference lists from relevant reviews, original research articles, consensus statements, and international guidelines. Studies published in non-peer-reviewed supplements were excluded. The final selection of references was based on their relevance to the mechanisms, epidemiology, biomarkers, and prevention of autoimmune thrombotic diseases.

Autoimmune diseases (AIDs) arise from inappropriate activation of the immune system leading to damage of otherwise healthy tissues and organs. As a result, virtually all AIDs have at least some inflammatory component and inflammation has a well-established prothrombotic effect. However, the autoimmune disorder with the closest relationship to thrombosis is antiphospholipid syndrome (APS), a disorder in which the autoantibodies have direct prothrombotic effects. Autoimmune-mediated thrombosis is an increasingly important challenge for European health systems. As populations age and survival with chronic autoimmune diseases improves, thrombotic complications contribute substantially to premature cardiovascular morbidity, disability, recurrent hospitalisation, pregnancy complications, and healthcare utilisation. Although autoimmune diseases affect a relatively small proportion of the population, their disproportionate contribution to cardiovascular disease in younger adults, particularly women, highlights the need for earlier recognition, improved risk stratification, and integration of autoimmune disorders into European cardiovascular prevention strategies.

Thrombosis is one of the diagnostic criteria for APS whose prevalence appears to be around 40–50 per 100,000 in western populations with a marked preponderance of females (4:1).^1^ Systemic lupus erythematosus (SLE) is very closely linked to APS and also associated with increased risk of thrombosis.^2^ Around 20–30% of patients with SLE have antiphospholipid antibodies (aPL)^3^ and of these ∼40% develop thrombosis making a diagnosis of secondary APS.^2^ Patients with SLE and APS carry significantly higher risk of thrombosis and recurrence compared to when they present in isolation.^2^ Other AIDs that are associated with increased risk of thrombosis and premature cardiovascular disease include rheumatoid arthritis (RA), large vessel vasculitis such as Takayasu's arteritis (TA) or Giant cell arteritis (GCA), medium vessel vasculitis such as Polyarteritis nodosa (PAN) and small vessel vasculitis such as ANCA associated vasculitis. In addition, vaccines (vaccine induced thrombocytopaenia and thrombosis [VITT]) or infections (coronavirus disease [COVID]-19 related thrombosis or VITT like syndrome) and drug-related immune reactions (heparin induced thrombocytopaenia [HIT]) can trigger profound disturbances in immune regulation and coagulation, resulting in thrombotic complications that resemble classical autoimmune thrombo-inflammatory syndromes. Table 1 summarises the reported incidence of these disorders, which represent a substantial public health burden.

**Table 1:** Reported incidence of antibody mediated autoimmune disorders commonly associated with thrombosis.

Spectrum of autoimmune disease	Incidence
Systemic Lupus Erythematosus (SLE)	20–150 cases per 100,000
Rheumatoid arthritis	0.5–1.0 cases per 100^4^
Large vessel vasculitis such as Takayasu's arteritis (TA) or Giant cell arteritis (GCA)	1–2 per million for TA. 7.3 per 100,000 for GCA
Medium vessel vasculitis such as Polyarteritis nodosa (PAN)	0.9–8 per million in Europe
Small vessel vasculitides such as ANCA associated vasculitis	24.7 per million in Norway
Antiphospholipid syndrome	Annual incidence of 1–2 per 100,000.^5^
Heparin induced thrombocytopaenia	The reported incidence ranges from 1 in 5000 to 3–7 in 100 inpatients depending on the patient population.^6^
Vaccine induced thrombocytopaenia and thrombosis (VITT)	Historically reported incidence of VITT following adenovirus-based COVID-19 vaccination is ∼1:100,000 among patients ≥50 years and at least 1:50,000 among patients in the younger group.^6^ True incidence of VITT like syndrome is unknown

ANCA = Anti-neutrophil cytoplasm antibodies.

Unlike heritable thrombophilias, thrombosis in these autoimmune antibody mediated diseases may occur in any vascular bed including venous, arterial or microvascular thrombosis although the most common presentation is venous thrombosis. For example, in APS, venous thromboembolism (VTE) occurs in 39% whilst stroke (19.8%) is the most common arterial thrombosis followed by transient ischaemic attack (11.1%) and myocardial infarction (MI) (5.5%).^7^ Furthermore, microvascular thrombosis and thrombosis at unusual sites such as cerebral venous sinus, splanchnic, retina are also seen in patients with APS. Although not the focus here, placental complications in pregnancy are an alternative qualifying criterion in APS. Notably the most frequent recurrent event in an APS cohort followed for 5 years was stroke, perhaps reflecting the limited efficiency of anticoagulant therapy in patients with APS.^8^ SLE, RA and other vasculitides are associated with increased risk of thrombosis, premature atherosclerosis and cardiovascular events. These complications arise for multiple reasons including disease or treatment related factors interacting with traditional vascular risk factors which are discussed in detail below. Due to their frequent association with stroke, myocardial infarction, venous thromboembolism and pregnancy complications, autoimmune thrombotic disorders contribute disproportionately to premature cardiovascular disease, disability and healthcare utilisation, making them an important but under-recognised public health challenge across Europe. In this narrative review, we discuss the pathophysiology, diagnosis, risk stratification, treatment and prevention both at individual-level and population-based levels of autoimmune mediated prothrombotic disease, focussing on APS as the archetypal example. HIT, VITT and COVID-19-associated thrombosis are not generally considered as autoimmune diseases but thrombosis in these conditions is driven by autoimmune processes and thrombosis in COVID-19 is multifactorial with immune processes playing a major role. Therefore, these conditions are also discussed with the same perspectives. It is important to emphasise that autoimmune-mediated thrombosis should be recognised not only as a mechanistic or disease-specific complication, but also as a preventable contributor to cardiovascular morbidity and premature mortality.

## Pathophysiology: from autoimmunity to thrombosis

The intricate interplay between innate immunity and coagulation enables the host to limit dissemination of circulating pathogens through thrombus formation. This coordinated protective mechanism, termed immunothrombosis, represents an evolutionary link between inflammation and haemostasis.^9^ However, excessive or dysregulated activation of these pathways during infection can shift this response toward pathological thrombo-inflammation which has been implicated in several conditions, including sepsis and autoimmune disorders such as APS and SLE. Hallmarks of thrombo-inflammation include endothelial dysfunction, platelet activation and aggregation, leucocyte adhesion and recruitment, neutrophil extracellular trap (NET) release and complement activation, promoting both arterial and venous thrombosis.^9^, ^10^, ^11^ In addition, thrombo-inflammation may involve direct interactions between autoantibodies, complement pathways, endothelial cells, neutrophils, platelets, and innate immune mechanisms.^9^^,^^12^ Increasing evidence suggests that thrombo-inflammation represents a central mechanism linking autoimmunity to cardiovascular morbidity and premature mortality.^13^

### Autoantibody mediated coagulation activation

Autoantibodies may have direct procoagulant effects and autoimmunity has general inflammatory and/or tissue damaging effects. Among the best characterised antibodies are anti-β2 glycoprotein I antibodies directed against phospholipid binding protein β2glycoprotein I causing a direct procoagulant effect^9^ in patients with APS and antibodies against heparin-platelet factor 4 (PF4) in HIT. aPL interact with phospholipid binding proteins on endothelial cells, monocytes, and platelets, leading to tissue factor (TF) expression, thrombin generation, and platelet aggregation.^14^^,^^15^ In APS, this process creates a sustained hypercoagulable state associated with arterial and venous thromboses, microvascular complications and pregnancy morbidities.^9^

Beyond APS and HIT, other autoimmune diseases also exhibit prothrombotic autoantibody profiles and in these conditions general inflammatory or tissue damaging effects cause the prothrombotic phenotype. In SLE, immune complexes and anti-double stranded DNA antibodies contribute to vascular inflammation and coagulation activation.^16^ Rheumatoid factor and anti-citrullinated protein antibodies in RA have similarly been associated with increased cardiovascular risk and endothelial activation.^17^ Chronic B-cell stimulation further amplifies inflammatory cytokine production, including tumour necrosis factor-α (TNF-α), interleukin-1 (IL-1), and interleukin-6 (IL-6), which enhance coagulation through upregulation of TF and suppression of natural anticoagulant pathways.^18^^,^^19^

Autoantibodies directed against protein S and protein C can induce acquired deficiencies/impaired function of these natural anticoagulants. Such antibodies have been reported in association with systemic autoimmune diseases including APS, infections, and post-infectious immune syndromes, and may present with catastrophic thrombotic manifestations including purpura fulminans, disseminated intravascular coagulation (DIC), and symmetrical peripheral gangrene.^20^^,^^21^ Immune complexes containing anti-histone antibodies have recently been implicated in thromboinflammatory conditions including severe COVID-19. These complexes can activate platelets through FcγRIIA signalling, promoting platelet aggregation, thrombin generation, and immunothrombosis.^22^ Increased awareness of these conditions among clinicians may facilitate earlier diagnosis and targeted treatment, particularly in patients with thrombosis occurring in unusual clinical contexts or in the absence of traditional thrombotic risk factors.

### Endothelial dysfunction

Under healthy physiological conditions, the vascular endothelium maintains antithrombotic and vasodilatory properties through production of nitric oxide, prostacyclin, and expression of anticoagulant proteins.^23^ Endothelial dysfunction arises in response to inflammation, tissue damage and direct binding of autoantibodies and is a pivotal step towards thrombosis, transforming the endothelium into a proinflammatory and prothrombotic surface.^24^

Inflammatory cytokines such as TNF-α, IL-1, and interferon-α promote endothelial activation by increasing expression of adhesion molecules, TF and procoagulant proteins including release of von Willebrand factor and down regulation of anticoagulant molecules.^25^ Activated endothelial cells facilitate leucocyte adhesion^26^ and platelet recruitment^27^ while reducing fibrinolytic capacity by upregulation of the main fibrinolytic inhibitor, plasminogen activator inhibitor 1 (PAI-1).^28^ Raised factor VIII and fibrinogen during inflammation also create a prothrombotic phenotype. Oxidative stress generated during chronic inflammation impairs nitric oxide bioavailability and accelerates vascular injury.^29^ In diseases such as SLE and RA, endothelial dysfunction contributes not only to thrombosis but also to accelerated atherosclerosis and premature cardiovascular disease.^30^

Endothelial injury may also result from direct immune-mediated mechanisms. Immune complex deposition, complement activation, and cytotoxic autoantibodies can damage the vascular lining and expose subendothelial collagen, thereby triggering platelet adhesion and coagulation activation.^31^ Vasculitic disorders provide a clear example of immune-mediated endothelial destruction leading to thrombosis and organ ischaemia.^32^ Additionally, chronic glucocorticoid exposure and disease-related metabolic disturbances may exacerbate endothelial dysfunction, further amplifying thrombotic risk.^33^

### Complement activation

Complement activation constitutes another critical link between immunity and thrombosis.^34^ Activation of the classical and alternative complement pathways promotes endothelial injury, leucocyte recruitment, and platelet activation. Complement fragments C3a and C5a induce inflammatory cytokine release and amplify thrombin generation, while the membrane attack complex directly damages endothelial cells.^35^ Experimental and clinical studies in APS demonstrate that complement activation is essential for thrombosis development, with complement inhibition emerging as a potential therapeutic strategy in severe or refractory disease.^36^ Furthermore, complement mediated microvascular thrombosis has been implicated in catastrophic APS and systemic vasculitis.^37^

### Cellular targets of autoimmunity

In AIDs, thrombosis can occur as a result of autoantibodies directly targeting cells. In Immune Thrombocytopenic Purpura (ITP), autoantibodies target platelet membrane glycoproteins such as GPIIb/IIIa or GPIb-IX. Despite causing thrombocytopaenia, patients with ITP have increased thrombosis risk which is multifactorial including microparticle formation and endothelial dysfunction, enhanced thrombin formation and treatment related factors interacting with patient related factors as with other autoimmune thrombotic disorders.^38^, ^39^, ^40^ In autoimmune Haemolytic Anaemia (AIHA), autoantibodies against red blood cells react with erythrocyte surface antigens, leading to haemolysis which can cause endothelial injury by Fe^3+^, release of procoagulant microparticles, complement activation, platelet activation, nitric oxide depletion, and inflammation. Antineutrophil antibodies are directed against neutrophil cytoplasmic antigens such as proteinase-3 (PR3) or myeloperoxidase (MPO), producing vasculitic disorders collectively called ANCA-associated Vasculitis, including granulomatosis with polyangiitis and microscopic polyangiitis.

Platelets are increasingly recognised as active immune and inflammatory mediators. Chronic inflammatory stimulation enhances platelet reactivity, aggregation, and interaction with leukocytes and endothelial cells.^41^ Activated platelets release pro-inflammatory mediators, microparticles, and thromboxane A2, all of which contribute to thrombus formation and vascular inflammation.^42^ Elevated platelet activation markers have been identified in APS, SLE, and RA, correlating with increased cardiovascular and thrombotic complications.^43^^,^^44^

A major mechanism underlying platelet-driven immunothrombosis is activation through the platelet FcγRIIa (Fc gamma receptor IIa).^45^ FcγRIIa is the only Fc receptor expressed on platelets and binds the Fc portion of IgG-containing immune complexes.^46^ When pathogenic antibodies form immune complexes and cross-link FcγRIIa on the platelet surface, intracellular signalling pathways involving Src family kinases, Syk kinase, and phospholipase Cγ2 are activated.^46^^,^^47^ This results in rapid platelet activation, granule secretion, integrin activation, and platelet aggregation.^47^ Activated platelets subsequently release procoagulant microparticles rich in phosphatidylserine, providing a catalytic surface for thrombin generation. They also express P-selectin, which promotes interactions with neutrophils and monocytes, leading to the formation of platelet–leucocyte aggregates.^48^^,^^49^ These interactions stimulate NET formation, further amplifying coagulation and vascular occlusion.^48^^,^^49^

In APS, aPL can directly activate platelets through FcγRIIa-dependent pathways, either by binding β2-GPI on platelet surfaces or through immune complex formation.^49^ Similar FcγRIIa-mediated activation is central to HIT, where PF4-heparin immune complexes trigger widespread platelet activation and thrombosis despite thrombocytopaenia.^6^^,^^45^

FcγRIIa signalling is central to VITT pathogenesis as well.^45^^,^^50^ *In vitro* studies demonstrate that serum from patients with VITT induces platelet aggregation via FcγRIIa, which can be blocked by inhibition of FcγRIIa providing one rationale for the use of high-dose intravenous immunoglobulin (IVIG), which inhibits Fcγ receptor-mediated platelet activation.^6^

Thus, platelet FcγRIIa represents a key molecular link between adaptive immunity and thrombosis and FcγRIIa signalling pathways are increasingly viewed as attractive therapeutic targets for preventing immune-mediated thrombotic disorders.^51^

Neutrophil extracellular traps (NETs) have emerged as another major cellular contributor to immuno-thrombosis. NETs are web-like structures composed of DNA, histones, and granular proteins released by activated neutrophils in response to inflammatory stimuli.^52^ Although NET formation serves antimicrobial functions, NETosis also promotes thrombosis by activating platelets, damaging endothelial cells, and providing a scaffold for fibrin deposition.^53^ In APS and SLE, impaired NET clearance and persistent neutrophil activation contribute to sustained vascular inflammation and coagulation activation.^54^^,^^55^ Histones and proteases released within NETs also enhance thrombin generation and inhibit endogenous anticoagulant mechanisms.^56^

### Interaction with traditional vascular risk factors

**Fig. 1:**
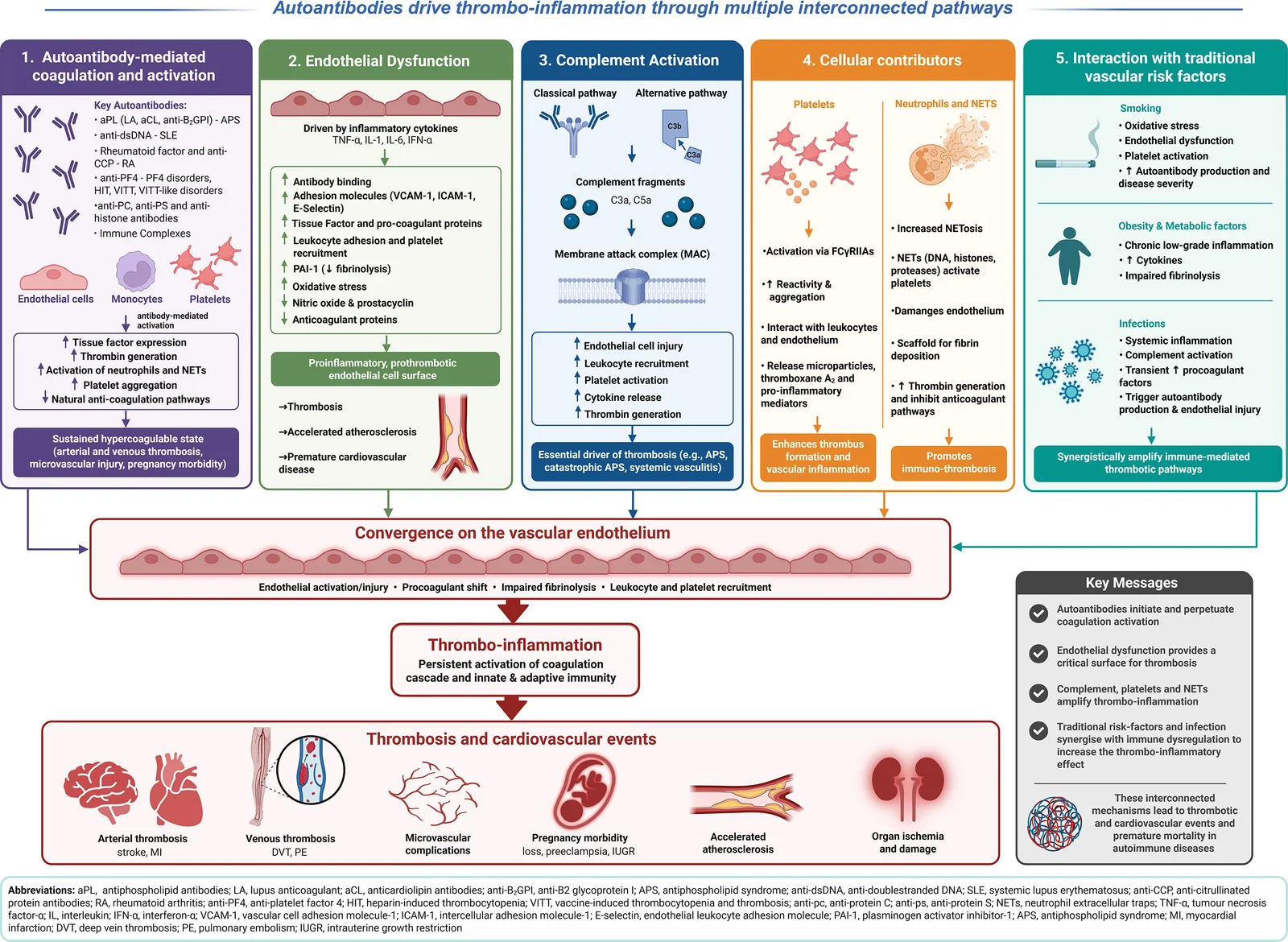
**From autoimmunity to thrombosis and cardiovascular events.** In autoimmune disease, thrombosis and cardiovascular complications occur through multiple interconnected pathways **(1)** Autoantibody-mediated coagulation activation through endothelial, monocyte, and platelet activation with increased tissue factor expression whilst downregulating naturally occurring anticoagulant leading to thrombin generation, and platelet aggregation. **(2)** Endothelial dysfunction, promoted by inflammatory cytokines (TNF-α, IL-1, IL-6, IFN-α), results in enhanced leucocyte adhesion, oxidative stress, impaired fibrinolysis, reduced nitric oxide and prostacyclin activity, and a prothrombotic endothelial phenotype. **(3)** Complement activation through classical and alternative pathways generates inflammatory mediators (C3a, C5a, membrane attack complex), promoting endothelial injury, platelet activation, leucocyte recruitment, cytokine release, and thrombin generation. **(4)** Cellular contributors including activated platelets including activation through FcγRIIa receptors and neutrophil extracellular traps (NETs) amplify thrombus formation, vascular inflammation, fibrin deposition, and immune thrombosis. **(5)** Traditional vascular risk factors such as smoking, obesity, metabolic dysfunction, and infections synergistically enhance immune-mediated thrombosis through oxidative stress, endothelial dysfunction, chronic inflammation, and complement activation. These pathways converge on the vascular endothelium, resulting in endothelial activation, impaired fibrinolysis, platelet and leucocyte recruitment, and persistent thrombo-inflammation. The downstream clinical consequences include arterial and venous thrombosis, microvascular complications, pregnancy morbidity, accelerated atherosclerosis, and organ ischemia/damage.

The thrombotic burden in autoimmune diseases is further amplified by traditional vascular risk factors. Smoking promotes oxidative stress, endothelial dysfunction, and platelet activation, while also increasing autoantibody production and disease severity in conditions such as RA and SLE.^57^ Obesity contributes to chronic low-grade inflammation, elevated cytokine levels,^58^ and impaired fibrinolysis, thereby compounding immune-mediated thrombotic pathways.^59^ Fig. 1 summarises the pathways from autoimmunity to thrombosis.

## Diagnosis and risk stratification

The diagnosis and risk stratification of thrombotic complications in autoimmune diseases remain clinically challenging because thrombotic risk is multifactorial, dynamic, and often underestimated by conventional cardiovascular assessment tools. Patients with autoimmune disorders may develop both arterial and venous thrombosis at younger ages and in the absence of traditional cardiovascular risk factors.

Clinical evaluation begins with comprehensive assessment of thrombotic history, disease activity, and cardiovascular comorbidities. A prior history of VTE, stroke, myocardial infarction, recurrent pregnancy loss, or microvascular thrombosis strongly increases future thrombotic risk in patients with APS.^60^

Laboratory investigations play a central role in identifying patients at increased risk especially APS. Testing for aPL, including lupus anticoagulant (LA), anticardiolipin antibodies, and anti-β2 glycoprotein I antibodies, is essential in patients with suspected APS or unexplained thrombotic events. Persistent positivity on repeat testing after at least 12 weeks confirms clinically significant aPL positivity and is associated with increased thrombotic recurrence risk.^61^ Triple positivity (positive for all aPL tests routinely used to diagnose APS), confers particularly high risk and is strongly associated recurrent thrombosis^62^ and obstetric complications.^63^

Markers of systemic inflammation such as C-reactive protein (CRP) and erythrocyte sedimentation rate (ESR) may indirectly reflect thrombotic propensity, especially in RA and SLE although ESR is no longer use in some countries as it is an unreliable marker of acute inflammation. Elevated complement consumption, particularly reduced C3 and C4 levels in SLE, may indicate active immune complex disease and endothelial injury. Additional biomarkers under investigation include D-dimer, circulating TF, platelet derived microparticles, and NET components, indicators of thrombo-inflammatory activity. Table 2 summarises the standard diagnostic tests for AID associated with thrombosis discussed here. However, detailed diagnostic assays on specific AIDs are outside the scope of this review.

**Table 2:** Routine diagnostic tests for autoimmune diseases associated with thrombosis.

Autoimmune disease	Key diagnostic tests
Antiphospholipid syndrome	Lupus anticoagulant, IgG and/or IgM anti-β2 glycoprotein I antibodies and anti-cardiolipin antibodies
Systemic Lupus Erythematosus	ANA, anti-dsDNA, anti-Sm antibodies, complement levels (C3/C4)
Immune Thrombocytopenic Purpura	Platelet count, peripheral blood smear, antiplatelet antibodies (limited use), diagnosis of exclusion for other causes of thrombocytopaenia
Autoimmune Haemolytic Anaemia	Direct antiglobulin (Coombs) test, LDH, bilirubin, haptoglobin
ANCA-associated vasculitis	c-ANCA (PR3), p-ANCA (MPO), ESR/CRP, biopsy
Rheumatoid Arthritis	Rheumatoid factor (RF), anti-CCP antibodies, ESR^a^/CRP
Heparin induced thrombocytopaenia	Detection of heparin platelet-factor 4 antibodies with ELISA or CLIA and confirmation assay demonstrating ability of these antibodies to activate platelets through functional assay (HIPA, LTA, SRA which are heparin dependent) or flowcytometry
Vaccine induced thrombocytopenia and thrombosis	Detection of platelet factor 4 antibodies with ELISA and confirmation with functional assay such as PIPA, LTA, PF4-enhanced SRA, PT, aPTT, D- dimer and Clauss fibrinogen to assist the diagnosis

ANA = anti-nuclear antibody; anti-dsDNA = anti-double standard DNA; LDH = lactate dehydrogenase; ANCA = Anti-neutrophil cytoplasm antibodies; PR3 = Anti-Proteinase 3 Antibody; MPO = Myeloperoxidase; ESR = erythrocyte sedimentation rate; CRP = C-reactive protein; anti-CCP = anti-cyclic citrullinated peptide; ELISA = Enzyme-linked immunosorbent assays; CLIA = Chemiluminescent immunoassays; HIPA = Heparin-induced platelet activation assay; PIPA = PF4-induced platelet activation assay; LTA = Light transmission aggregometry; SRA = Serotonin-release assay.

a ESR is no longer use in some countries as it nonspecific and it may not reflect the acute inflammation.

Imaging studies are important both for diagnosis of acute thrombosis and assessment of subclinical vascular disease. In autoimmune populations, vascular imaging may identify asymptomatic atherosclerosis, arterial stiffness, or endothelial dysfunction. Carotid ultrasound and coronary artery calcium scoring have demonstrated increased prevalence of premature atherosclerosis in SLE and RA patients compared with the general population.^64^

Risk stratification requires integration of autoimmune-specific and traditional cardiovascular risk factors. Conventional tools such as the Framingham Risk Score or SCORE2 (Systematic Coronary Risk Evaluation 2), a tool to estimate 10-year risk of fatal and non-fatal cardiovascular disease in adults without prior CVD or diabetes may underestimate cardiovascular risk in inflammatory diseases.^65^ This is because they do not account for chronic systemic inflammation or immune mediated vascular injury. Consequently, several rheumatology societies recommend multiplication factors or disease specific adjustments when estimating cardiovascular risk in autoimmune rheumatic diseases.

Traditional risk factors including smoking, hypertension, obesity, diabetes mellitus, dyslipidaemia, and sedentary lifestyle should be systematically evaluated, as they synergistically increase thrombotic burden in autoimmune disease. Medication commonly used in autoimmune diseases such as glucocorticoids may worsen metabolic and vascular risk.^33^

Overall, effective diagnosis and risk stratification require a multidisciplinary approach combining clinical history, serological markers, imaging, and assessment of inflammatory activity. Improved identification of high-risk patients may facilitate earlier preventive interventions and personalised thromboprophylaxis strategies in autoimmune disease populations.

## Treatment and prevention: individual-level interventions

Because thrombosis in autoimmune disorders arises through interactions between immune dysregulation, endothelial dysfunction, and coagulation activation, optimal management extends beyond conventional anticoagulation alone.^9^ Individual level interventions therefore involve a combination of antithrombotic therapy, immunomodulation, and aggressive control of cardiovascular risk factors. Treatment approaches must be individualised according to disease subtype, thrombotic history, antibody profile, bleeding risk, and overall inflammatory burden.

### Anticoagulation

Anticoagulation remains the cornerstone of treatment for venous and arterial thrombotic events in autoimmune diseases, particularly in APS. Vitamin K antagonists (VKA) have historically been the standard long-term therapy for thrombotic APS, with intensity guided by thrombotic phenotype and recurrence risk.^66^ Patients with recurrent thrombosis or arterial events may require higher-intensity anticoagulation or combination with antiplatelet treatment.^66^ Lifelong anticoagulation is often recommended in individuals with persistent aPL and thrombotic events because recurrence rates are high after treatment discontinuation.^66^

Direct oral anticoagulants (DOACs) have transformed management of VTE in the general population. However, a prospective randomised controlled trial (RCT) with high-risk (triple-positive) APS, demonstrated increased rates of recurrent thrombosis, especially arterial thrombosis (stroke and myocardial infarction), with rivaroxaban compared with warfarin.^67^ Subsequent meta-analysis including four RCTs showed that patients with triple positive aPL receiving DOACs had a significantly higher rate of recurrence arterial thrombosis, irrespective of the type of index thrombotic event (venous or arterial).^68^ There was a trend towards higher recurrence of arterial thrombosis in patients with single or dual positive aPL.^68^ Consequently, current guidelines recommend against the use of DOAC in high-risk APS.^61^ Nevertheless, selected low-risk patients (single or dual positive) intolerant to warfarin may still be considered for DOAC therapy after careful risk assessment.^61^

Low dose aspirin is widely used for primary thromboprophylaxis in high-risk autoimmune populations, particularly in SLE patients and individuals with aPL but no prior thrombosis as per European Alliance of Associations for Rheumatology (EULAR) guidelines^69^ although this is not recommended by the British Society for Haematology (BSH).^61^ BSH guidelines suggest use of thromboprophylaxis for asymptomatic aPL carriers in high-risk situations, avoidance of oestrogen containing contraceptive pills and systemic hormone replacement therapy.^69^ In obstetric APS, low dose aspirin combined with prophylactic low molecular weight heparin substantially improves maternal and foetal outcomes.^69^

Cardiovascular risk reduction remains crucial alongside anticoagulation. Smoking cessation, blood pressure, weight, diabetes and lipid control, statin therapy, regular exercise, and avoiding prolonged immobility may significantly reduce thrombotic risk. There is growing evidence that statins exert both anti-inflammatory and antithrombotic effects.^70^

### Immunomodulation

Because chronic inflammation is a major driver of thrombosis in autoimmune disease, immunomodulatory therapy plays a central role in prevention strategies.^9^ Effective suppression of disease activity reduces endothelial dysfunction, cytokine-mediated coagulation activation, and platelet hyperreactivity. In RA and SLE, better inflammatory control has been associated with lower cardiovascular and thrombotic event rates.^71^

Hydroxychloroquine is one of the most important immunomodulatory agents with antithrombotic properties, particularly in SLE and APS. In addition to reducing disease activity, hydroxychloroquine inhibits platelet aggregation, decreases aPL mediated endothelial activation, and improves lipid and glucose metabolism.^72^ Observational studies suggest that hydroxychloroquine use is associated with reduced thrombotic events and improved survival in lupus populations.^72^

Biologic therapies targeting inflammatory cytokines such as TNF-α, IL-6 and B cells may also contribute to vascular protection. TNF inhibitors in RA have been associated with improved endothelial function and lower cardiovascular risk compared with uncontrolled disease. Rituximab and complement inhibitors are increasingly explored in refractory APS and catastrophic APS, where immune-mediated thrombosis is severe and rapidly progressive.^70^

Vitamin D has important immunomodulatory properties and has been implicated in the pathogenesis of several autoimmune disorders including APS,^9^ RA and SLE.^73^ Low vitamin D levels may contribute to immune response dysregulation.^73^ Given the low risk associated with supplementation, vitamin D replacement is commonly recommended for APS patients with deficiency (serum vitamin D < 50 nmol/L), provided there are no contraindications such as hypercalcaemia, typically at 800–1000 IU daily.

Glucocorticoids remain important for acute disease control but are prothrombotic and may contribute to hypertension, dyslipidaemia, obesity, and insulin resistance when used long term.^33^ Minimising cumulative steroid exposure is therefore critical in reducing cardiovascular and thrombotic complications. Overall, prevention of thrombosis in autoimmune diseases requires a multidisciplinary and individualised approach combining anticoagulation, targeted immunosuppression and cardiovascular risk reduction.

## Population level prevention

Given the elevated risk of premature cardiovascular morbidity and mortality in autoimmune diseases, public health approaches must focus on early identification of high-risk individuals, optimisation of modifiable risk factors, and integration of cardiovascular prevention into routine rheumatologic care/haematology care. Reducing the burden of autoimmune thrombosis will require coordinated clinical pathways that span primary, secondary and tertiary care, ensuring timely diagnosis, cardiovascular risk assessment, implementation of preventive therapies, and long-term follow-up.

Cardiovascular risk reduction strategies form the foundation of population level prevention. Smoking cessation programmes, promotion of physical activity, healthy dietary interventions, and obesity prevention are particularly important because traditional cardiovascular risk factors synergistically amplify immune mediated vascular injury. Aggressive management of hypertension, diabetes mellitus, and dyslipidaemia should be prioritised in autoimmune populations, with regular monitoring incorporated into disease management pathways. Vaccination programmes and infection prevention strategies may also reduce inflammatory triggers. Embedding cardiovascular prevention within routine autoimmune disease management, rather than treating it as a separate speciality responsibility, may improve adherence to preventive therapies and reduce long-term vascular complications.

Screening in high-risk groups is another key preventive strategy. Patients with persistent aPL, prolonged disease duration, high inflammatory burden, lupus nephritis or previous pregnancy morbidity may benefit from enhanced cardiovascular and thrombotic surveillance. Routine assessment of blood pressure, lipid profiles, glucose metabolism and smoking status should be integrated into specialist clinics and primary care settings. Selected patients may also benefit from vascular imaging techniques, including carotid ultrasonography or coronary artery calcium scoring, to detect subclinical atherosclerosis. Standardised cardiovascular assessment at diagnosis and at regular intervals thereafter could be incorporated into clinical pathways alongside disease activity monitoring.

Importantly, conventional cardiovascular risk prediction tools often underestimate risk in autoimmune diseases. Greater awareness among healthcare professionals of this pitfall and implementation of disease-specific screening protocols are required to improve early prevention and reduce long-term vascular complications: see Table 3. Future cardiovascular risk assessment models should incorporate autoimmune disease characteristics, including disease duration, inflammatory burden, autoantibody profile and cumulative immunosuppressive exposure, to improve prediction and guide preventive interventions.

**Table 3:** Prevention strategies for autoimmune disease related cardiovascular events.

Prevention area	Population-level recommendation	Target group	Expected benefit
Cardiovascular risk reduction	Integrate cardiovascular prevention into routine rheumatology and haematology care pathways	All patients with autoimmune disease	Reduces premature cardiovascular morbidity and mortality
Smoking cessation	Provide structured smoking cessation programmes and counselling	Smokers with autoimmune disease	Reduces endothelial injury, thrombosis, and atherosclerosis risk
Physical activity promotion	Encourage regular exercise through public health and clinical interventions	Autoimmune disease populations	Improves cardiovascular health and reduces inflammation
Healthy diet and obesity prevention	Promote healthy diets and weight management programmes	General autoimmune populations	Reduces metabolic risk factors contributing to vascular disease
Hypertension management	Regular blood pressure monitoring and aggressive treatment	High-risk autoimmune patients	Lowers risk of stroke, myocardial infarction, and vascular damage
Diabetes prevention and control	Routine glucose screening and glycaemic optimisation	Patients with metabolic risk factors	Reduces vascular and thrombotic complications
Dyslipidaemia management	Regular lipid screening and treatment with statin	Autoimmune disease populations	Reduces accelerated atherosclerosis risk
Vaccination and infection prevention	Improve vaccination uptake and infection control measures Choose non-adenovirus vaccines whenever available	Immunosuppressed and high-risk patients	Reduces inflammatory triggers for thrombotic events
Early access to disease-modifying therapy	Improve availability and adherence to DMARDs/immunosuppressive therapies	Patients with active autoimmune disease	Suppresses chronic inflammation and endothelial dysfunction
High-risk patient screening	Identify patients with antiphospholipid antibodies, lupus nephritis, prolonged disease duration, or high inflammatory burden	High-risk autoimmune subgroups	Enables earlier detection of vascular and thrombotic complications
Routine cardiovascular monitoring	Integrate blood pressure, lipid, glucose, and smoking assessments into long-term care	Specialist and primary care settings	Supports early intervention for modifiable risk factors
Vascular imaging surveillance	Use carotid ultrasound or coronary artery calcium scoring in selected high-risk patients	Patients with suspected subclinical atherosclerosis	Detects early vascular disease before clinical events occur
Disease-specific risk assessment	Develop and implement autoimmune disease-specific cardiovascular screening tools	Healthcare systems and clinicians	Improves accuracy of cardiovascular risk prediction
Healthcare professional education	Increase clinician awareness of underestimated cardiovascular risk in autoimmune disease	Rheumatology, haematology, and primary care professionals	Improves early prevention and long-term outcomes

Effective prevention therefore requires multidisciplinary models of care involving rheumatologists, haematologists, cardiologists, neurologists, nephrologists, obstetricians, vascular specialists, primary care physicians, specialist nurses and pharmacists. Shared-care pathways, supported by harmonised referral criteria and electronic health records, could facilitate coordinated risk assessment, treatment optimisation and surveillance, while reducing fragmentation of care.

All forms of thrombosis have significant public health and economic implications. The most common thrombosis is VTE which in 2014 was estimated to incur direct medical costs of $15,000–$20,000 per case in the USA with a total conservative estimated cost to the US healthcare system of $7–$10 billion each year.^74^ Although less frequent, stroke can result in greater disability and days of lost work but there is a wide variation in severity. The total cost of stroke in 32 European countries was estimated as €31 billion in 2017, of which €27 billion (85%) was due to healthcare spending.^75^ In both cases the contribution from autoimmune disorders is likely to be disproportionately large due to the younger age of patients and the effects of comorbidities, emphasising the importance of AIDs related thrombosis prevention.

From a policy perspective, autoimmune diseases should be recognised within broader non-communicable disease (NCD) and cardiovascular prevention strategies. National cardiovascular prevention programmes and clinical guidelines should explicitly identify autoimmune diseases as high-risk conditions, while quality indicators and clinical registries should include cardiovascular outcomes in patients with chronic inflammatory diseases. Improved integration of autoimmune diseases into prevention frameworks, together with investment in multidisciplinary services, implementation research and equitable access to preventive care, offers an important opportunity to reduce premature cardiovascular morbidity, healthcare utilisation and avoidable mortality across Europe. Table 4 summarised the clinical and public-health implications for different clinical disciplines such rheumatology, haematology, cardiovascular, primary care, multidisciplinary, policymakers and researchers.

**Table 4:** Key clinical and public health implications.

• Autoimmune diseases should be recognised as important cardiovascular risk-enhancing conditions. • Thrombotic complications contribute disproportionately to premature cardiovascular morbidity, disability and mortality, particularly among younger adults and women. • Cardiovascular prevention should be embedded within routine rheumatology and haematology care rather than delivered separately. • Conventional cardiovascular risk prediction tools frequently underestimate risk in autoimmune diseases; disease-specific assessment strategies are needed. • High-risk patients should undergo structured cardiovascular and thrombotic surveillance integrated across primary and specialist care. • Multidisciplinary care pathways involving rheumatologists, haematologists, cardiologists, neurologists, nephrologists, obstetricians and primary care can improve prevention and long-term outcomes. • Health systems should integrate autoimmune diseases into cardiovascular disease and broader non-communicable disease prevention strategies. • Education of primary care physicians to recognise early signs of autoimmune disease and ensure long-term monitoring, adherence to preventive therapies and timely referral for specialist assessment. • Future research should prioritise autoimmune-specific risk prediction, implementation of preventive care pathways and evaluation of cost-effective population-level interventions.

## Infection and drug induced autoimmunity and thrombosis

Increasing evidence demonstrates that dysregulated immune activation following viral infection, vaccination, or medication exposure can induce autoantibody formation, platelet activation, endothelial dysfunction, and complement-mediated thrombosis.^76^

The COVID-19 pandemic particularly highlighted the close relationship between inflammation, autoimmunity, and thrombosis,^76^ while conditions such as VITT and HIT have provided important mechanistic insights into immune-mediated coagulation disorders.^6^

### Links with COVID-19 and thrombosis

Early waves of COVID-19 were strongly associated with both venous and arterial thrombotic complications, including deep vein thrombosis, pulmonary embolism, stroke, myocardial infarction, and microvascular thrombosis.^77^ Early in the pandemic, patients with severe acute respiratory syndrome coronavirus 2 (SARS-CoV-2) infection were found to exhibit marked hypercoagulability, elevated D-dimer levels, thrombocytopaenia, and diffuse endothelial injury. Severe COVID-19 is now recognised as a thrombo-inflammatory disease in which excessive immune activation contributes directly to coagulation dysregulation.^77^

Thrombosis in COVID-19 is multifactorial in which systemic inflammation associated with Viral infection leads to inflammatory cytokine release, enhanced tissue factor expression and thrombin generation.^77^ Elevated levels of IL-6, TNF-α, and other inflammatory mediators amplify platelet activation and suppress fibrinolysis. NETs also play a major role by providing a scaffold for fibrin deposition and platelet aggregation.^77^

Importantly, SARS-CoV-2 infection has been associated with transient autoantibody production, including aPL.^77^ Although the clinical significance of these antibodies remains incompletely understood, their presence suggests overlap between infection-driven thrombo-inflammation and autoimmune thrombosis.^77^

### VITT and VITT-like syndromes

VITT emerged as a rare but serious complication following adenoviral vector COVID-19 vaccination. VITT is characterised by thrombosis occurring in association with thrombocytopaenia, typically developing within 5–30 days after vaccination. Unusual thrombotic sites, including cerebral venous sinus thrombosis and splanchnic vein thrombosis, are common and may be associated with severe morbidity and mortality.^6^

The pathogenesis of VITT resembles autoimmune HIT (see below). Patients develop IgG antibodies against PF4, induced by adenovirus of the vaccine which activate platelets through FcγRIIa receptor signalling in the absence of heparin exposure.^6^ VITT occurs in individuals carrying the immunoglobulin light-chain alleles IGLV3-21∗02 or ∗03 when specific somatic hypermutation develops in antibodies directed against a defined epitope of the adenoviral core protein pVII.^78^ This alteration redirects antibody binding toward PF4, leading to the pathogenic immune response characteristic of VITT.^78^ There is intense platelet activation, thrombin generation, and consumptive thrombocytopaenia. Complement activation and NET formation may further amplify thrombosis.^6^

Recognition of VITT significantly improved following development of diagnostic algorithms incorporating thrombocytopaenia, elevated D-dimer levels, low fibrinogen, and positive anti-PF4 antibody testing.^6^ Early treatment with non-heparin anticoagulants and high-dose intravenous immunoglobulin substantially improved outcomes.^6^ Corticosteroids and plasma exchange may be considered in severe or refractory cases.^6^

In addition to classical VITT which is no longer a clinical concern due to omission of adeno-virus vector-based vaccination, VITT-like syndromes have been reported with adenovirus infection and, rarely, following non-adenoviral vaccinations or without identifiable triggers.^6^ Another VITT like entity is monoclonal gammopathy of thrombotic significance which occurs in association with monoclonal paraproteinemia.^79^ Clinicians need to be aware of these rare but highly prothrombotic syndromes in patients presenting with thrombosis and thrombocytopaenia especially in association with very high D-dimers.^6^^,^^79^ These observations suggest that strong inflammatory stimuli may occasionally provoke pathogenic anti-PF4 immune responses independent of heparin exposure or without additional polyanions.

### HIT and thrombosis

HIT is one of the best characterised examples of drug-induced immune thrombosis. HIT occurs when exposure to polyanions, mainly heparin, triggers the formation of antibodies against PF4–heparin complexes.^6^ These immune complexes activate platelets through Fc receptor signalling, leading paradoxically to thrombosis despite thrombocytopaenia. Both venous and arterial thromboses may occur, including deep vein thrombosis, pulmonary embolism, limb ischaemia, and stroke.^6^

The risk of HIT varies according to heparin type and clinical setting, with unfractionated heparin carrying higher risk than low molecular weight heparin.^80^ Diagnosis relies on clinical assessment using scoring systems such as the 4T score combined with laboratory detection of anti-PF4 antibodies and functional platelet activation assays.^6^^,^^80^

Management requires immediate discontinuation of all heparin products and initiation of alternative anticoagulants.^80^ Delayed recognition can result in catastrophic thrombotic complications and high mortality. HIT has also provided a mechanistic framework for understanding autoimmune thrombotic syndromes such as VITT, illustrating how antibody-mediated platelet activation can bridge innate immunity, coagulation, and thrombosis.^80^

## Research gaps and future directions

Despite advances in understanding, important gaps remain in risk prediction, disease mechanisms, and treatment strategies. Current evidence is largely based on observational studies, retrospective analyses, and disease-specific cohorts. Large prospective studies are needed to better define the incidence, timing, and predictors of thrombosis across autoimmune diseases, populations, and geographic regions. This would help identify high-risk patients, evaluate long-term outcomes, and clarify interactions between autoimmune disease and traditional cardiovascular risk factors. Linking cohorts with biobanks and electronic health records could accelerate biomarker discovery, validation, and mechanistic research. Existing markers, including aPL, D-dimer, and inflammatory indices, have limited predictive accuracy and may not be applicable across different autoimmune thrombotic disorders. Emerging biomarkers such as complement activation products, neutrophil extracellular trap components, platelet-derived microparticles, interferon signatures, and endothelial dysfunction markers may improve risk stratification. Integration of biomarker, genomic, and proteomic data could enable personalised prediction models, while improved antibody testing remains a key research priority in APS, VITT-like syndromes, and other autoimmune thromboses.

Further clinical trials investigating targeted immunotherapies are also required. While anticoagulation remains central to thrombosis management, increasing evidence suggests that suppression of immune-mediated inflammation may substantially reduce thrombotic risk. Trials evaluating complement inhibitors, B-cell–directed therapies, cytokine inhibitors, and NET-targeting strategies may identify novel approaches for preventing recurrent thrombosis in high-risk autoimmune populations.

Antigen-specific therapies aim to selectively suppress harmful autoimmune responses without causing generalised immunosuppression. These therapies target disease-causing autoantigens, autoreactive T cells, or pathogenic antibodies. CD19-targeted CAR-T therapy is emerging as a promising treatment for autoimmune diseases including SLE.^81^ However, aPL often persist despite immunosuppression, suggesting involvement of long-lived plasma cells and alternative B-cell activation pathways.^9^ Bispecific antibodies (bsAbs) enable simultaneous targeting of multiple immune pathways involved in autoantibody production and thrombo-inflammation.^82^ Unlike conventional monoclonal antibodies, bsAbs can engage two distinct targets, such as CD3 and CD19/CD20 or B cell maturation antigen (BCMA), thereby promoting selective depletion of autoreactive B cells and plasma cells responsible for pathogenic antibody generation.^82^ BCMA-directed bispecific antibodies, such as teclistamab and linvoseltamab, can directly target plasma cells and therefore offer a theoretical advantage in eliminating the source of pathogenic aPL.^82^ Similarly, CD20 × CD3 bispecific T-cell engagers (mosunetuzumab, glofitamab, epcoritamab) induce profound depletion of autoreactive B cells and may achieve more complete immune resetting than conventional anti-CD20 monoclonal antibodies.^83^ This strategy is particularly appealing in patients with recurrent thrombosis despite anticoagulation, rituximab, or other immunosuppressive therapies.

Finally, development of international and multidisciplinary global registries would enhance data harmonisation, facilitate collaborative research, and improve understanding of rare syndromes such as catastrophic APS and VITT/VITT like syndrome.

## Policy and practice recommendations

The growing recognition of thrombosis as a major complication of autoimmune diseases highlights the need for coordinated policy and clinical practice reforms at both national and international levels. Autoimmune-associated thrombosis contributes substantially to cardiovascular morbidity, disability, healthcare utilisation, and premature mortality, yet considerable variation exists in diagnosis, risk assessment, and preventive care across healthcare systems. Strengthening global strategies for early identification and management is therefore essential.

One major priority is the development and implementation of standardised diagnostic criteria and risk assessment frameworks. Autoimmune thrombotic diseases often demonstrate heterogeneous clinical manifestations, resulting in delayed diagnosis and inconsistent management. Harmonised international criteria incorporating clinical, serological, and imaging findings would improve diagnostic accuracy, facilitate earlier intervention, and enhance comparability across clinical studies and registries.

Regular cardiovascular and thrombotic risk assessment, including screening for hypertension, dyslipidaemia, diabetes mellitus, smoking, obesity, and aPL, should become an integral component of rheumatology and primary care services. Early vascular imaging and inflammatory monitoring may help identify subclinical disease before overt thrombotic complications occur. Educational programmes aimed at healthcare professionals and patients are equally important to improve awareness of thrombotic symptoms, preventive strategies, and the importance of long-term disease control.

Integration of autoimmune-associated thrombosis into broader non-communicable disease (NCD) frameworks represents another important policy direction. Current cardiovascular prevention programmes often overlook chronic inflammatory and autoimmune conditions despite their strong association with premature vascular disease. Incorporating autoimmune disorders into national cardiovascular and NCD action plans would improve care models, access to preventive therapies and encourage inclusion of inflammatory diseases in population-level cardiovascular surveillace.

## Conclusions

Autoimmune thrombosis represents a critical intersection between immunology, inflammation, and cardiovascular disease. Increasing evidence demonstrates that chronic immune dysregulation contributes directly to endothelial injury, coagulation, platelet and neutrophil activation, complement-mediated thrombo-inflammation, and accelerated atherosclerosis, thereby substantially increasing the risk of arterial and venous thrombotic events. Conditions such as SLE, RA, APS, and vasculitis exemplify how persistent inflammation and autoantibody production transform the vascular system into a prothrombotic environment. The emergence of thrombo-inflammatory syndromes associated with COVID-19, VITT, and HIT has further strengthened understanding of the complex links between immunity and thrombosis.

Beyond their effects on individual patients, autoimmune thrombotic disorders represent an under-recognised contributor to premature cardiovascular morbidity, disability, and healthcare utilisation across Europe and globally. Despite advances in diagnosis and treatment, thrombotic complications remain a major cause of recurrent vascular events and premature mortality in patients with autoimmune diseases. There is therefore an urgent need for prevention-focused strategies that integrate earlier risk stratification, aggressive control of inflammation, cardiovascular risk reduction, and personalised antithrombotic therapy within multidisciplinary models of care. Incorporating autoimmune diseases into cardiovascular prevention frameworks and health-system planning offers an important opportunity to reduce avoidable morbidity, healthcare burden, and premature mortality.

## Contributors

Deepa J Arachchillage (DJA) conceptualised and designed the manuscript. DJA and Mike Laffan (ML) wrote and revised the manuscript. Megan V Preece, (MVP) drew the figure designed by DJA and revised the manuscript. All authors approved the final version of the manuscript.

## Declaration of interests

DJA received funding from Werfen, Stago and Sysmex to attend national and international meetings and received a speaker fee from Werfen, California. DJA is the chair of the International Society for Haemostasias and thrombosis (ISTH) Scientific and Standardization Committee (SSC) subcommittee on antiphospholipid syndrome. MVP and ML declare no conflicts of interest.
